# Vibrio ecology, pathogenesis, and evolution

**DOI:** 10.3389/fmicb.2014.00256

**Published:** 2014-05-28

**Authors:** Daniela Ceccarelli, Rita R. Colwell

**Affiliations:** ^1^Department of Cell Biology and Molecular Genetics, Maryland Pathogen Research Institute, University of MarylandCollege Park, MD, USA; ^2^Department of Environmental Health Sciences, Johns Hopkins Bloomberg School of Public Health, Johns Hopkins UniversityBaltimore, MD, USA

**Keywords:** Vibrio, ecology, genome, evolution, pathogenesis

This Research Topic brings together 24 articles that highlight the most recent research findings concerning the biology of the genus *Vibrio* and covers pathogenicity and host interaction, genome plasticity and evolution, and the dynamics of factors influencing the ecology of vibrios.

*Vibrio* comprises one of the most diverse marine bacterial genera (Gomez-Gil et al., 2014), and its diversity is emphasized in two of the articles opening this set of Research Topic papers. Sawabe et al. (2013) present a molecular phylogeny of 86 *Vibrio* species based on genome sequencing that provides insight into *Vibrio* biodiversity and evolutionary history. In a study of more than 300 *Vibrio* genome sequences, Lukjancenko and Ussery (2014) conclude that the *Vibrio* pan-genome comprises 17,000 gene families, differentially present and/or expressed in any given species.

A remarkable feature of all *Vibrio* species is an highly plastic genome, a feature examined in five papers. The two chromosomes are shaped by horizontal gene transfer involving, among others, antibiotic resistance, virulence, and niche adaptation (Rowe-Magnus et al., 2001; Kirkup et al., 2010). *V. vulnificus* biotype 3 is a notable example. Efimov et al. (2013) suggest biotype 3 evolved from biotype 1 by acquisition of unique genes from other bacterial species, such as *Shewanella*, sharing the same ecological niche. Carraro et al. (2014) employ molecular and functional characterization of pVCR94, to identify the role of IncA/C plasmids in antibiotic resistance in a Rwandan *V. cholerae* isolate. A retrospective analysis of epidemic *V. cholerae* in Angola is reported by Valia et al. (2013), showing unexpected genomic variability among variants, highlighting the role of genomic islands, phages, and integrative conjugative elements in the genetic diversity of *V. cholerae* in a single epidemic. Rivas et al. (2013) describe acquisition by *Photobacterium damselae* subsp. *damselae* of virulence plasmid pPHDD1 that encodes pore-forming toxins and hemolysins which play a key role in virulence for both fish and humans. A review by Rapa and Labbate (2013) describes the role of integrons in *Vibrio* species for which gene cassettes comprise approximately 1–3% of the entire genome and are very likely involved in bacterial adaptation and evolution.

Nine of the manuscripts analyze *Vibrio* pathogenicity, disease development, specificity, and adaptation in both human and animal hosts. Tan et al. (2014) deciphered the biosynthetic network of the siderophore vulnibactin, essential in iron uptake from host proteins, the importance of which in *V. vulnificus* pathogenicity has been clinically demonstrated. Inhibition/resistance mechanisms developed by *V. salmonicida*, the causative agent of hemorrhagic septicemia in Atlantic salmon, is described by Bjelland et al. (2013), who show that it overcomes the salmon innate immune system to a point where the infection overwhelms the host. The role in bacterial virulence of diverse extracellular proteolytic enzymes secreted by human pathogenic *Vibrio* species is the focus of a review by Miyoshi (2013). The engagement of type VI secretion systems by *V. cholerae* is suggested as a means of intra- and inter-species predation and nutrient acquisition, inducing rapid multiplication in the human host (Pukatzki and Provenzano, 2013). The bioluminescent marine bacterium *V. campbellii* is used by Wang et al. (2013) to analyze the pyomelanin-pigmented phenotype, known to provide *Vibrio* species with greater UV and oxidative stress resistance and enhanced intestine colonization. The relationship between pathogenicity and motility in *Vibrio* species and the contribution of flagella to adhesion and biofilm formation are discussed by Zhu et al. (2013). The largely unexplored *V. fluvialis* mechanisms of pathogenesis, survival and fitness are reviewed by Ramamurthy et al. (2014). Twenty new *Vibrio* species associated with molluscans are described and their pathogenic potential for molluscs elucidated by Romalde et al. (2014). The exquisite bacteria–host interaction between *V. fisheri* and its squid host, *Euprymna scolopes*, is described in detail, as are the molecular pathways of biofilm formation, motility, and chemotaxis (Norsworthy and Visick, 2013).

The capacity of *Vibrio* species to persist in the aquatic environment, their ecology and association with abiotic and biotic factors, as well as environmental surveillance for public health (Lipp et al., 2002; Grimes et al., 2009; Johnson, 2013) comprise a section in the Research Topic that opens with a review by Lutz et al. (2013) elucidating complex mechanisms enabling *V. cholerae* to withstand starvation, temperature fluctuation, salinity variation, and predation. Haley et al. (2014) report water temperature increase can be correlated with rise of a diverse population of *V. parahaemolyticus*, some of which carry pandemic markers, in water and plankton along the Georgian coast of the Black Sea. *V. parahaemolyticus* and *V. vulnificus* populations associated with oyster, sediment, and surface water related to a hurricane event in the Chesapeake Bay are concluded to be influenced by wave energy and sediment resuspension (Shaw et al., 2014). Canesi et al. (2013) show the serum of *Mytilus galloprovincialis* promotes phagocytosis and killing by hemocytes of both *V. cholerae* O1 and non-O1/non-O139 in edible bivalves. Chakraborty et al. (2013) evaluate a sensitive and specific dipstick test to detect toxigenic *V. cholerae* in water, validating a simple, inexpensive method for use in areas at risk of cholera.

Three articles addressing *Vibrio* environmental diversity and dynamics complete this Research Topic. Mansergh and Zehr (2014) suggest that the natural shift of *Vibrio* populations in Monterey Bay is affected by larger oceanographic conditions (flow velocities and wind patterns), rather than individual environmental factors. Meta-analysis of environmental variables and *Vibrio* association with plants, algae, zooplankton, and animals are reviewed by Takemura et al. (2014). As a final point concerning environmental distribution, Constantin De Magny et al. (2014) propose temporal shifts, zooplankton community variability, and occurrence of *V. cholerae* in the aquatic environment are related to cholera dynamics. These factors, analyzed by metagenomics, permit greater understanding of community structure, function, and competition.

In summary, the collection of manuscripts provided in this Research Topic offers a comprehensive exploration of *Vibrio* biology, from the single gene to the bacterial community, elucidating *Vibrio* molecular pathways and evolutionary history. This special issue shows the significant progress achieved in understanding the complex biology of the genus *Vibrio* and should both stimulate discussion and offer a challenge to researchers in microbial ecology and evolution.

## Conflict of interest statement

The authors declare that the research was conducted in the absence of any commercial or financial relationships that could be construed as a potential conflict of interest.
